# Statistical characterization of the GxxxG glycine repeats in the flagellar biosynthesis protein FliH and its Type III secretion homologue YscL

**DOI:** 10.1186/1471-2180-9-72

**Published:** 2009-04-16

**Authors:** Brett Trost, Stanley A Moore

**Affiliations:** 1 Department of Computer Science, University of Saskatchewan, 110 Science Place, Saskatoon, S7N 5C9, Canada; 2 Department of Biochemistry, University of Saskatchewan, 107 Wiggins Road, Saskatoon, S7N 5E5, Canada

## Abstract

**Background:**

FliH is a protein involved in the export of components of the bacterial flagellum and we herein describe the presence of glycine-rich repeats in FliH of the form AxxxG(xxxG)_*m*_xxxA, where the value of *m *varies considerably in FliH proteins from different bacteria. While GxxxG and AxxxA patterns have previously been described, the long glycine repeat segments in FliH proteins have yet to be characterized. The Type III secretion system homologue to FliH (YscL, AscL, PscL, etc.) also contains a similar GxxxG repeat, and hence the presence of the repeat is evolutionarily conserved in these proteins, suggesting an important structural role or biological function.

**Results:**

A set of FliH and YscL protein sequences was downloaded from GenBank, and then filtered to reduce redundancy, to ensure the soundness of the sequences, and to eliminate, as much as possible, confounding phylogenetic signal between individual sequences by implementing a pairwise 25% sequence identity cut-off. The general features of the glycine-rich repeats in these proteins were examined, and it was found that the length of these repeat segments varied substantially among FliH proteins but was fairly consistent for the Type III (YscL) homologue sequences, with values of *m *ranging from 0 to 12 for FliH and 0 to 2 for YscL. The amino acid sequence distribution of each of the three positions in the GxxxG repeats was found to differ significantly from the overall amino acid composition of the FliH/YscL proteins. The high frequency of Glu, Gln, Lys and Ala residues in the repeat positions, which is not likely indicative of any contaminating phylogenetic signal, suggests an α-helical structure for this motif. In addition, we sought to determine whether certain pairs of amino acids, in certain pairs of positions, were found together significantly more often than would be predicted by chance. Several statistically significant correlations were uncovered, which may be important for maintaining helical stability or for forming helix-helix interactions. These correlations are likely not of a phylogenetic origin as the originating sequences for the pair correlations are derived from a low similarity set and the individual incidences of the pair correlations do not cluster in any obvious phylogenetic sense, nor is there much evidence of strict sequence conservation outside the positions of the glycine residues. Finally, the α-helices from a non-redundant set of proteins from the Protein Data Bank were searched for GxxxG repeats similar in length to those found in FliH, however there were no helices containing more than three contiguous glycine repeat segments; thus, long glycine repeats similar to those found in FliH are presumably quite rare in nature.

**Conclusion:**

The glycine repeats in YscL and particularly FliH represent an intriguing amino acid sequence motif that is very rare in nature. Although we do not attempt to offer a mechanism whereby these repeats may have evolved, we do place the existence of the motif and some residue pairings within a rational structural context. While crystal structures of these proteins are necessary to fully elucidate the structural and functional significance of these repeats, the characterization reported here represents a first step in understanding this unique sequence feature.

## Background

The bacterial flagellum is an apparatus that projects outward from the cell membrane, and employs rotation of a flexible filament attached to a universal joint (the hook) for propulsion. The flagellum is made up of four components: the basal body, which houses the flagellar rotary motor and export apparatus; the rod, which spans the periplasm, peptidoglycan, and outer membrane; the hook, which acts as a universal joint; and the filament, which acts as the propulsion device (reviewed in [1,2]). In order to construct a functional flagellum, the constituent proteins must first be synthesized in the cytoplasm and then be transported to their site of incorporation in a temporally and spatially regulated manner. A specialized Type III secretion system called the flagellar export apparatus is used to transport the individual components of the flagellum across the two cell membranes of gram-negative bacteria [1]. The bacterial flagellar export apparatus (reviewed in [1,2]) is composed of a number of proteins, including two integral membrane proteins FlhA and FlhB, that also contain globular cytoplasmic domains, four additional integral membrane proteins FliO, FliP, FliQ, and FliR, and two membrane-associated cytoplasmic proteins, FliH and FliI. Other structural components of the flagellar basal body (FliF), and C-ring (FliG, FliM, FliN) are also required for flagellum assembly. In addition, enteric gram-negative bacteria have a number of substrate-specific chaperones associated with the flagellar export apparatus (e.g. FlgN, FliT, FliS, FliJ). These proteins act in concert with the flagellar export ATPase FliI in translocating partially unfolded substrates, such as the filament component flagellin, in an export-competent state through the basal body pore. Ultrastructural and biochemical investigations of the flagellar basal body and the Type III secretion system indicate that these systems have evolved from a common ancestor [3,4]. In support of these observations, most of the flagellar export components have conserved orthologues (ranging from 20–40% pairwise identity) in the Type III secretion system of gram-negative pathogenic bacteria [5,6], including FliI (InvC, HrcN etc.), FliH (YscL), FliN (HrcQ_B_), and FlhA (SctV) [7-11].

**Figure 1:**
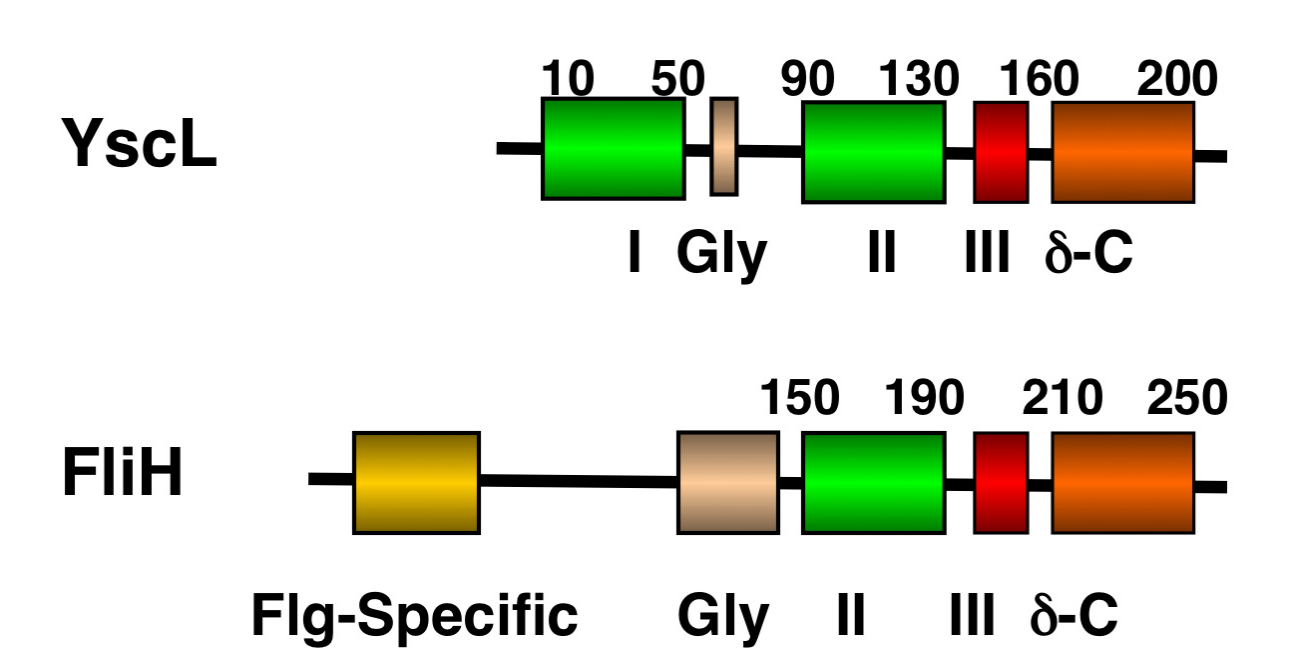
**Primary Sequence of FliH and YscL – schematic representation of domain organization in FliH and YscL proteins**. A flagellum specific region at the N-terminus of FliH which has no correspondence to YscL is shown in gold. An N-terminal YscL-unique segment is shown in green and labelled I. The glycine rich segments described in the text are coloured gold and labelled Gly. The green segment labelled II corresponds to a segment in FliH and YscL homologues found to be similar to the F_1 _ATPase b-subunits [21]. The red segment labelled III is unique to FliH and YscL. The orange segment labelled δ-C is proposed by Pallen and co-workers to be homologous to the delta subunit (AtpF) of F_1 _ATPase [21].

**Figure 2:**
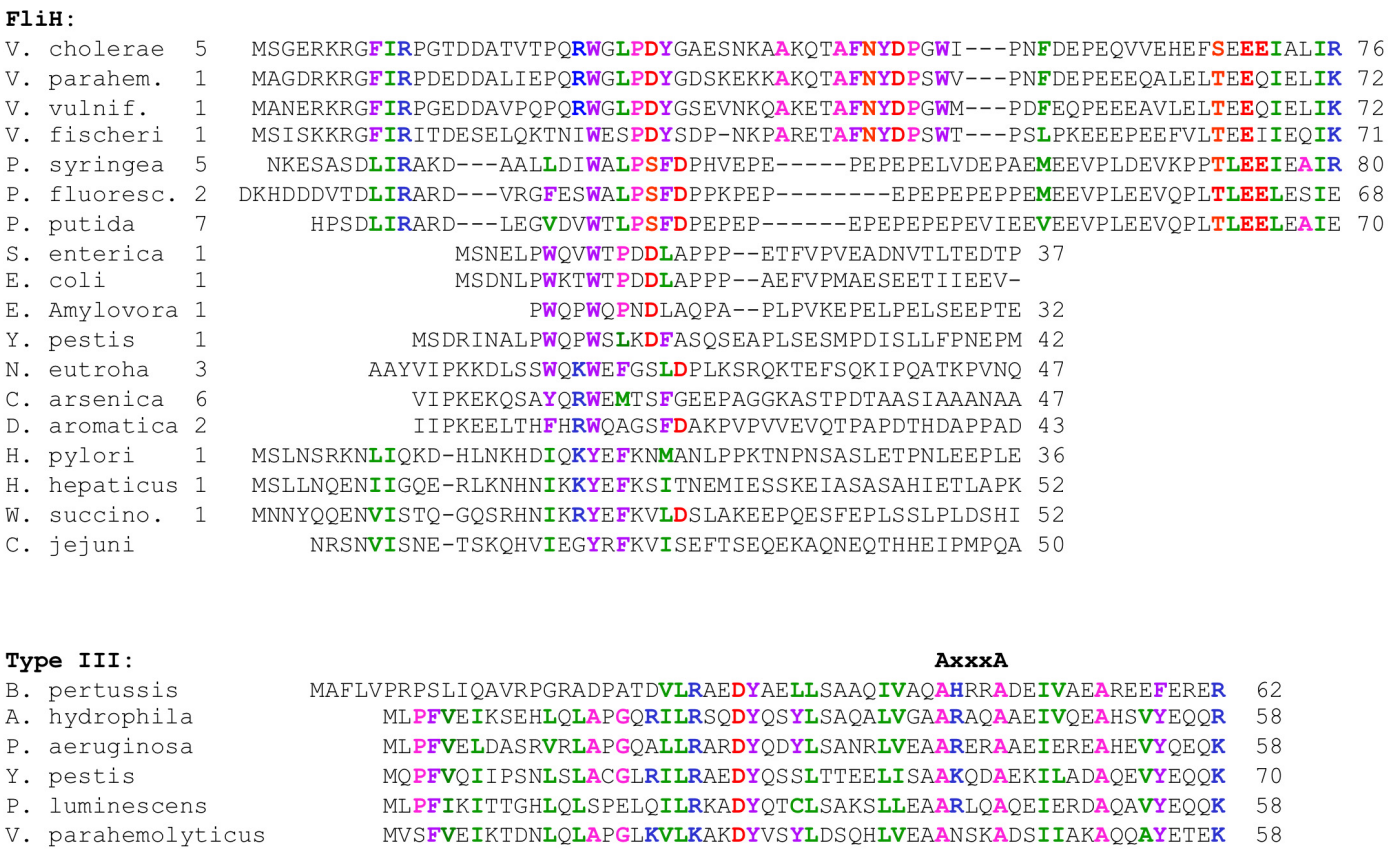
**Primary Sequence of FliH and YscL – alignment of the N-terminal sequences of FliH from a number of bacterial groups that exhibit weak conservation of primary sequence**. The unrelated segment at the N-terminus of YscL is shown for comparison.

**Figure 3:**
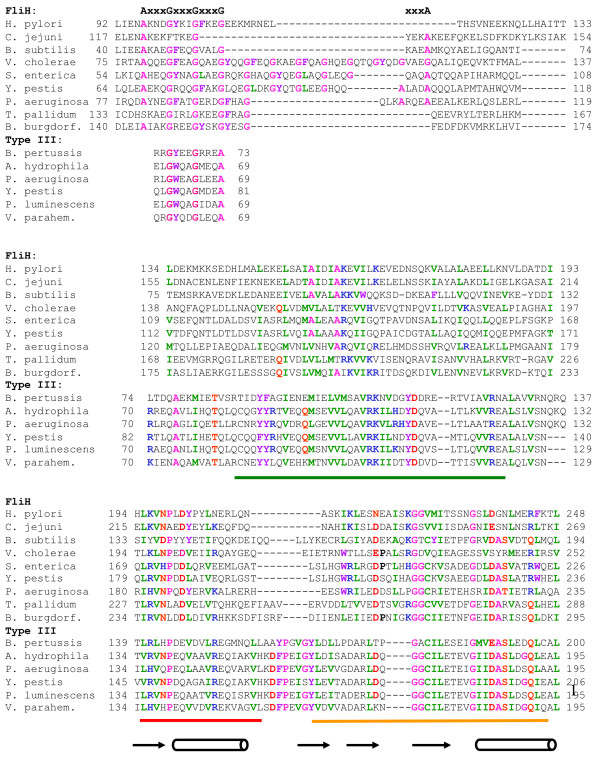
**Primary Sequence of FliH and YscL – multiple alignment of the C-terminal conserved region of FliH and YscL showing the position of the AxxxG(xxxG)_*m*_xxxA repeats for some representative sequences**. Coloured bars relate the sequence segments denoted as II (green), III (red) and δ-C described in Figure 1. Secondary structure prediction for the globular domain at the C-terminus of FliH/YscL is shown as arrows and cylinders for beta strands and alpha helices respectively. Predictions calculated using [35-39].

Functions and molecular interactions similar to their flagellar counterparts have been demonstrated for some of the Type III export proteins (e.g. InvC to FliI, HrcQ_B _to FliN, YscL to FliH) [7-13], and are generally assumed for the other components. For example, the *Salmonella *and *H. pylori *FliH proteins have been shown to interact with the highly conserved FliI ATPase [12-18] and the flagellar rotor C-ring protein FliN is also known to interact with FliH in *Salmonella *[9,13]. In Type III secretion systems, the FliH homologue (e.g. YscL) has been shown to interact specifically with the respective FliI homologue (e.g. YscN), as well as the corresponding FliN homologue, HrcQ_B _[7-9,12]. *Salmonella *FliH forms an elongated dimeric structure in solution [16,18], and forms a (FliH)_2_FliI complex [16]. Residues 100–235 of *Salmonella *FliH are required for interaction with FliI, residues 101–141 of FliH are required for FliH dimerization, and FliH N-terminal residues contribute to binding to the enterobacterial flagellar chaperone FliJ [17]. In addition residues spanning amino acids 60–100 of FliH appear important for inhibition of FliI ATPase activity as deletion of residues 60–100 enhances FliI ATPase activity *in vitro *[17]. Furthermore, deleting either residues 70–80 or 90–100 of *Salmonella *FliH reduce the magnitude of FliI ATPase inhibition [17]. However, it is unclear how amino acids spanning residues 60–100 of *Salmonella *FliH affect FliI ATPase activity, although inhibition appears to be non-competitive in the related Type III system [19]. Furthermore, a conserved AxxxG(xxxG)_*m*_xxxA motif, which is the focus of this report, spans residues 59–94 in *Salmonella *FliH (Figures 1, 2 and 3), suggesting that these FliH GxxxG repeats may have a role in FliI ATPase regulation. In addition, the precise role of FliH in flagellar protein secretion is not presently understood. A recent study examining the motility of bacteria with mutant flagellar proteins found that FliI-null mutants are non-motile, FliH-null mutants are weakly motile, and, interestingly, that FliI/FliH double mutants displayed greater (but still impaired) motility than FliI-null mutants after extended incubation [20]. Motivated by the realization that the mode of interaction between FliI and FliH is strikingly similar to that of the N-terminal α-helix of the F_1 _ATPase α-subunit with the globular domain of the F_1 _ATPase δ-subunit [18], we have previously suggested that FliH may function as a molecular stator in combination with FliI during the export of flagellum components [18]. In support of this idea, we and other researchers have noted weak but significant sequence similarity between FliH/YscL and the b-subunit of F_o_F_1 _ATPases ([7,21]; S. Moore, unpublished results).

The present study investigates a conserved GxxxG (where "x" represents any amino acid) sequence motif unique to the flagellar FliH/YscL family of proteins. Naming conventions for YscL-like proteins are rather inconsistent, as this protein often has different names in different organisms; for ease of reference, all YscL-like proteins will be referred to in this paper simply as "YscL". An alignment of the complete sequences of a representative group of FliH and YscL sequences along with a schematic domain organization is provided in Figures 1, 2 and 3. The extreme N-terminal region of FliH is very poorly conserved, but some sequence conservation is evident in the various bacterial groups (e.g. enterobacteria, epsilon proteobacteria), but not the YscL protein family. A GxxxG segment of variable length follows, then a poorly conserved segment likely to be helical in structure, followed by a well-conserved C-terminal domain known to be responsible for the interaction with the N-terminus of the flagellar/Type III ATPase (Figures 1, 2 and 3).

When we noticed the presence of conserved consecutive GxxxG repeats in FliH/YscL, we asked if this motif had been previously observed in other types of proteins. Lemmon *et al*. [22] first discovered that specific interactions are required for the transmembrane helix-helix dimerization of glycophorin A. It was later shown that dimerization was mediated by a GxxxG-containing motif [23]. The GxxxG motif has been identified as the dominant motif in the transmembrane regions of hundreds of proteins [24,25], and appears to play a critical role in the stabilization of helix-helix interactions. Such motifs were subsequently observed in many soluble proteins [26]. The amino acid composition of the variable positions in the glycine repeats of soluble proteins is certain to be very different from that of transmembrane proteins; transmembrane proteins would contain mostly hydrophobic residues in the variable positions of the repeats, while the variable positions in soluble proteins would contain mostly hydrophilic residues. As such, the only commonality between glycine repeats in transmembrane proteins and glycine repeats in soluble proteins is likely to be the glycines found at every fourth residue. As glycine lacks a side chain, it is suitable for allowing the close packing of helices, and could hence facilitate helix-helix dimerization.

Most annotated FliH sequences contain a segment of repeats of the form AxxxG(xxxG)_*m*_xxxA, where *m *can vary on average between 2 and 10 depending on the bacterial species. While there is some variation to this pattern, not all sequences contain the N-terminal-side Axxx or the C-terminal-side xxxA, and FliH proteins from some species have no GxxxG repeats at all. Nevertheless, a significant proportion (44% in our set of sequences) of FliH proteins extracted from the non-redundant sequence database (see Methods) do exhibit the AxxxG(xxxG)_m_xxxA pattern. In addition to this long AxxxG(xxxG)_*m*_xxxA repeat segment, most FliH proteins also contain one or more shorter repeat segments elsewhere in the primary sequence (Figures 1, 2 and 3), which usually contain just a single AxxxG, GxxxG, or GxxxA. These shorter repeat segments are very poorly conserved, do not contain an obvious preference for particular amino acids at any of the three middle non-glycine positions, and often contain proline. Hence, these non-conserved GxxxG segments are unlikely to be either helical or biologically significant. To differentiate the two patterns, we will refer to the longest repeat segment in a particular FliH protein as its "primary repeat segment". YscL proteins exhibit similar patterns, except that they generally have shorter primary repeat segments.

We report here a statistical characterization of the amino acids composing the variable positions in the primary repeat segments of a varied collection of FliH and YscL sequences from different bacterial species. As they are analyzed separately, the specific portion of the repeat segments being discussed – AxxxG, GxxxG, or GxxxA – will be referred to as the "repeat type". Additionally, we make the distinction between the first, second, and third variable residue in a given repeat, which will be denoted as positions x_1_, x_2_, and x_3_, respectively. Below, we describe the analysis performed on FliH, which is of primary interest due to its uniquely long primary repeat segments. Some of the analysis described below was also performed for YscL; full details are provided in the Results and Methods sections.

**Figure 4:**
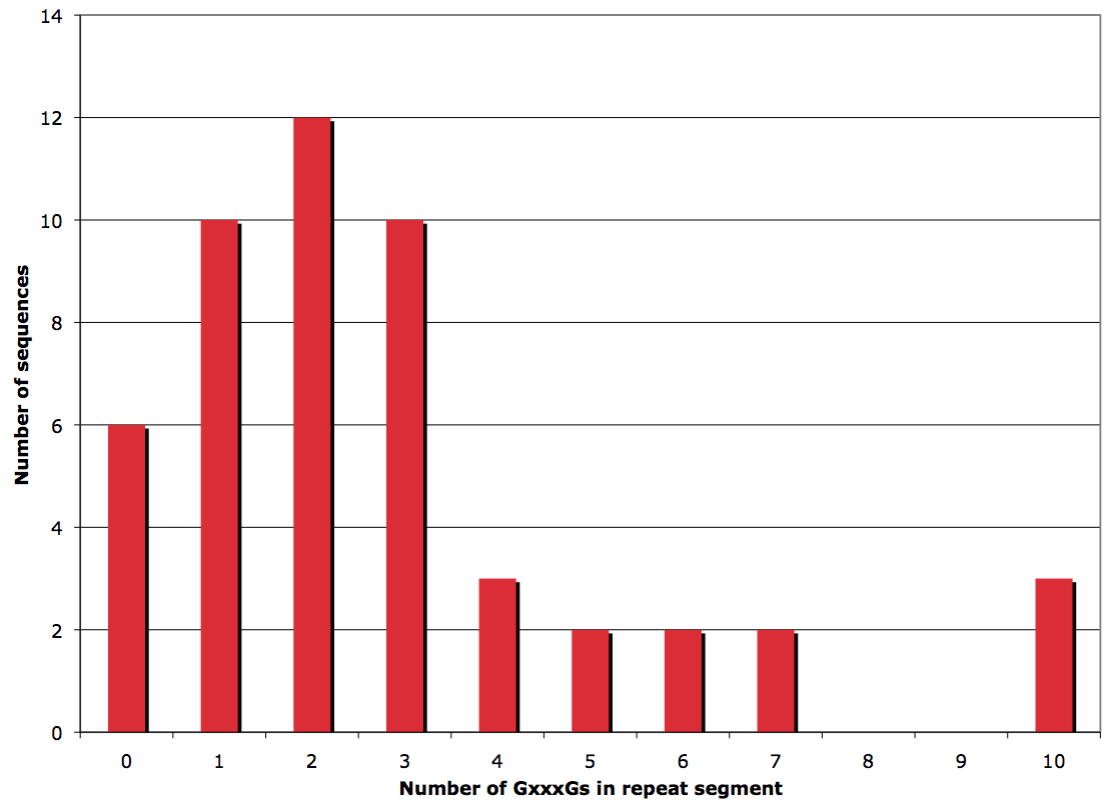
**Number of FliH sequences having primary repeat segments of different lengths**. The number of FliH sequences having primary repeat segments of different lengths is shown. The number on the x-axis represents only the number of GxxxGs; flanking AxxxGs and GxxxAs were not counted.

**Figure 5:**
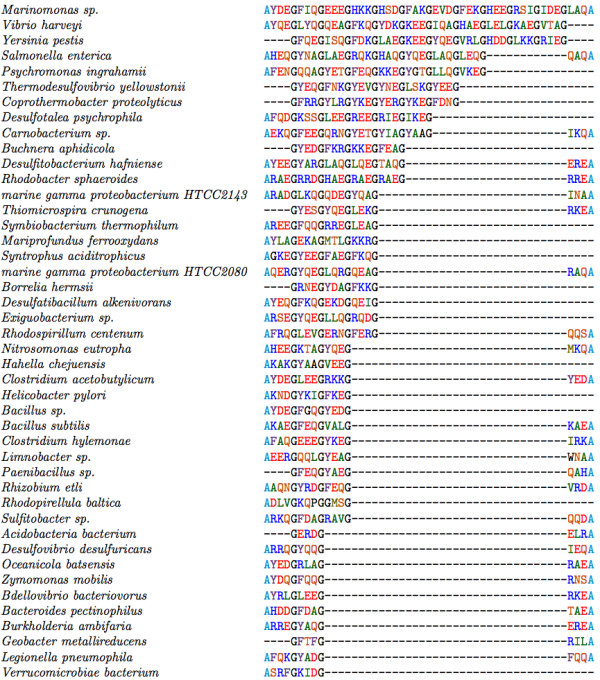
**Multiple alignment of the primary repeat segments from the FliH proteins of different organisms**. The primary repeat segments in the FliH proteins were aligned by hand. Only sequences that contained a repeat segment appear in this alignment.

To provide a general characterization of the glycine repeats in FliH, some initial data were gathered, such as the number of proteins having a repeat segment flanked by Axxx and xxxA, and the lengths of the primary repeat segments in each sequence. Next, secondary structure prediction programs were employed to predict whether the glycine repeat segments are likely to adopt a helical conformation, as would be expected given the amino acid compositions of these repeats, as well as previous results concerning the role of glycine repeats in helix-helix dimerization. A multiple alignment of the glycine repeat segments of FliH and YscL was then created, which provides insight into how FliH/YscL proteins from different bacterial species relate to each other in terms of the length and composition of their primary repeat segments. The distribution of amino acids in the three variable positions in each repeat type was then determined. We hypothesized that the amino acid frequencies in the glycine repeats would differ significantly from the amino acid frequencies in the entirety of all the FliH/YscL proteins; to provide support for this hypothesis, statistical tests were used to determine the probability that any differences found could have occurred by chance. To ensure that the tabulated amino acid frequencies and positional correlations were not simply the result of high sequence similarity due to sampling sequences that are phylogenetically closely related (especially in the GxxxG segment), we employed an overall 25% amino acid sequence identity cut-off to filter out highly similar FliH sequences and select an approximately even sampling of the available FliH sequences. This results in very little observable sequence similarity throughout the aligned FliH sequences that were ultimately selected for the analysis (essentially no absolutely conserved residues and only a few highly conserved residues, see Additional files 1 and 2). For the GxxxG motif region, there is always going to be evidence of phylogenetic signal due to the strongly conserved glycine residues (30.7% identical for GxxxGxxxGxxxG) and there is certainly some conservation in the lengths of the repeats in sequences that are more closely related (Figures 4 and 5). However, the imposed 25% sequence identity cutoff in our data analysis has filtered most of the apparent sequence similarity in the variable regions of the repeat. This can be seen by comparing the similarity between any two aligned sequences both within the repeat region (Figure 5) and outside of the repeats (see Additional files 1 and 2). For FliH, we calculated correlation coefficients between all possible pairs of amino acids, in all possible combinations of positions in the repeats, and used statistical methods to determine whether certain pairs of amino acids in specific positions are found together significantly more often than would be expected by chance. We hypothesized that certain pairs of amino acids in nearby positions, such as positions within the same repeat, or in adjacent repeats, would be highly correlated, while amino acids in positions farther away from each other would be unlikely to be strongly correlated, and that the correlations are due to selective pressure imposed by structural constraints on the GxxxG motifs. For instance, in α-helices, there is a well known incidence of oppositely charged residues (for example glutamate and lysine) occurring in i, i+4 or i, i+3 pairs, therefore forming stabilizing intra-helical salt bridges, and these are typically not highly conserved interactions. Rather they appear to be the result of random mutations and selective pressures to stabilize nearby charged residues within the context of the helical structure. Similar results have been found for pair correlations in β-sheets [37].

Finally, we sought to determine how prevalent long glycine repeats are in other types of proteins not related to FliH, and to identify a protein of known three-dimensional structure that contains a FliH-like repeat segment that is involved in helix-helix dimerization. To address both goals, a large number of protein structures were downloaded from the Protein Data Bank (PDB; http://www.rcsb.org/pdb). These structures were searched for the presence of helices with glycine repeats, and one protein with a FliH-like glycine repeat segment was chosen as a molecular model for the types of interactions that might occur in FliH proteins.

The work presented here represents a comprehensive characterization of a relatively unusual primary sequence pattern. While this study focuses mainly on FliH/YscL and their glycine repeat segments, the results should also add to our understanding of the general characteristics of glycine repeat-containing α-helices in water-soluble proteins.

## Results

### Sets of proteins acquired

FliH proteins and YscL proteins were downloaded and filtered as described in the Methods section to obtain a set of FliH sequences and a set of YscL sequences where no sequence was more than 25% identical to any other sequence. After filtering, 50 FliH sequences and 16 YscL sequences remained.

### Initial characterization of glycine repeat segments

Initially, some general data regarding the composition of the 50 chosen FliH sequences were gathered. The average number of GxxxGs found in a primary repeat segment was 2.84, with a standard deviation of 2.53; the fewest number found in this set was 0, while the greatest number was 10. (In describing the length of a sequence's primary repeat segment, we include only GxxxGs; AxxxGs and GxxxAs are not included in the total). Although the longest repeat found in this dataset was 10, there exist FliH sequences with even longer repeats. For instance, the FliH from *E. coli *strain 53638 (GenBank accession number EDU66533) contains a repeat of length 12; however, this sequence was excluded when imposing the 25% identity sequence cut-off. A histogram showing the number of FliH sequences having primary repeat segments of different lengths is given in Figure 4. The majority of sequences have repeats with a length of 3 or less, while a few sequences have much longer repeats. Interestingly, the distribution of the lengths of the primary repeat segments in a set of 167 FliH sequences for which no sequence is more than 90% identical to any other sequence is very similar to that shown in Figure 4, indicating that bias arising from high sequence similarity in the available FliH sequences used has little effect on the results. This histogram is available as Additional file 3. In contrast to FliH, the primary repeat segments of YscL were much more uniform in length. Five sequences had no repeat segment at all, while 7 sequences had a repeat of length 1 and 4 sequences had a repeat of length 2. This stark difference in the distribution of the repeat lengths between FliH and YscL invites speculation concerning the importance of the repeat in these two proteins. As FliH apparently experiences selection pressure for longer repeats, but YscL does not, it suggests that longer repeats are advantageous to the function of FliH, but not to YscL; however, the nature of this difference is unclear.

Of the FliH sequences that had at least one GxxxG (a total of 44 sequences), the repeat segments of 22 sequences were flanked by both an Axxx on the N-terminal side and an xxxA on the C-terminal side. A lower number (13 sequences) contained only an initial Axxx, while few sequences had only an xxxA at the end (4 sequences) or neither an N-terminal-side Axxx nor a C-terminal-side xxxA (5 sequences). It thus appears that the initial Axxx is more strongly conserved than the terminating xxxA. Just two of the YscL sequences contained repeats with both the initial AxxxG and the terminal GxxxA, and an equal number (4 each) contained only the initial AxxxG or only the terminal GxxxA.

### Secondary structure prediction

Several secondary structure prediction programs were used to predict the secondary structure of the primary repeat segments of selected FliH and YscL proteins, and the prediction programs consistently and convincingly classified these regions as α-helical for all of the proteins tested. The tools used are given in [27-31]. Thus, there is a strong basis for interpreting the sequence characteristics of the glycine repeat segments as being important either for helical stability, or for making helix-helix interactions.

### Multiple alignment of the glycine repeats

**Figure 6:**
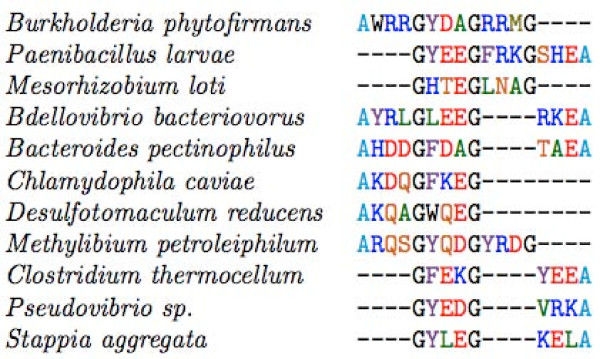
**Multiple alignment of the primary repeat segments from the YscL proteins of different organisms**. The primary repeat segments in the YscL proteins were aligned by hand. Only sequences that contained a repeat segment appear in this alignment.

We have performed a multiple alignment of the glycine repeats in both FliH (Figure 5) and YscL (Figure 6) to illustrate the composition of their repeat segments. The alignment was essentially carried out by hand and forces both the initial (Axxx or Gxxx) and terminal (xxxA or xxxG) motif to be in the same register. One interesting observation in Figure 5 is that sequences with shorter repeats appear to be more likely to have the initial Axxx and the terminating xxxG than sequences with longer repeats, suggesting that longer repeats may compensate in some way for the absence of the alanine "caps".

### Calculating the amino acid distribution in the primary repeat segments

**Figure 7:**
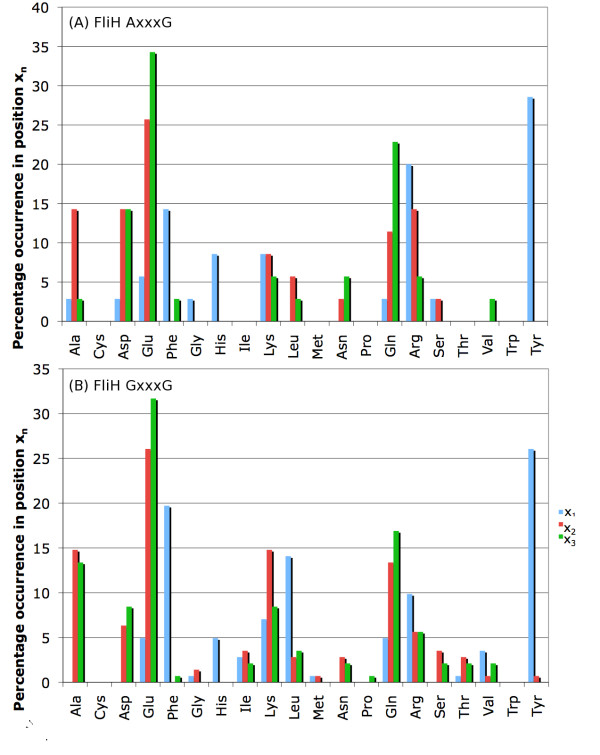
**Amino acid distribution of the primary repeat segments (part 1)**. The frequency of each amino acid in each position (x_1_, x_2_, and x_3_) of the FliH proteins are shown for AxxxGs (A) and GxxxGs (B).

**Figure 8:**
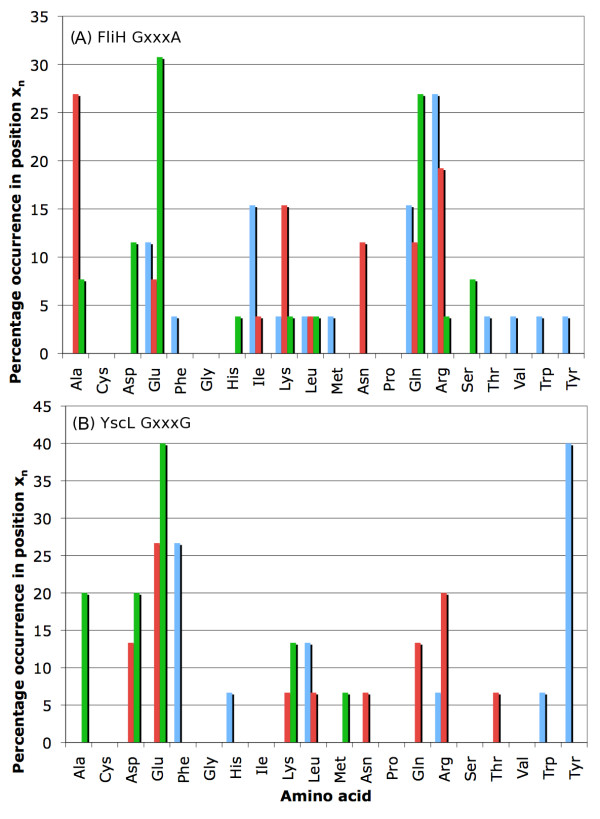
**Amino acid distribution of the primary repeat segments (part 2)**. The frequency of each amino acid in each position (x_1_, x_2_, and x_3_) of the FliH proteins are shown for GxxxAs (A). In addition, the amino acid distribution for GxxxGs in YscL is given in (B).

After this initial characterization of the glycine repeats, we then sought to determine the frequency of each amino acid in each position of each repeat type. Figures 7 and 8 give these data for all three repeat types in FliH, and just for GxxxGs in YscL (the sample size of AxxxGs and GxxxAs in YscL is too small to justify making inferences about the distribution of amino acids in the variable positions). While the frequencies reported in Figures 7 and 8 certainly appear to diverge significantly from what one might consider to be a "normal" distribution of amino acids, we confirmed this observation statistically. A χ^2 ^test was used to determine whether the amino acid frequencies in each position – repeat-type combination was significantly different than the amino acid frequencies in the entirety of all the FliH proteins. The x_1_, x_2_, and x_3 _positions in both AxxxGs and GxxxGs all had P-values less than 10^-30^, while those same positions for GxxxAs had P-values of 1.4 × 10^-3^, 1.8 × 10^-9^, and 9.0 × 10^-17 ^respectively. For YscL, the P-values for all three variable positions in the GxxxG repeats were less than 10^-29 ^(again, we do not comment on the distribution of the variable positions in YscL AxxxGs and GxxxAs due to the small sample size). Thus, it can readily be seen that the amino acid distribution in the primary repeat segments is significantly different than the overall composition of the FliH/YscL sequences. Moreover, it is unlikely these frequencies are simply the product of phylogenetic signal as the sequence similarity between the proteins in the dataset is minimal, especially in the variable residues of the GxxxG repeats (the glycine residues notwithstanding), rather we suggest that the observed amino acid frequencies at x_1_, x_2 _and x_3 _more likely are the result of selective pressure arising from helical structural constraints imposed by the GxxxG motif and its possible structural role in FliI ATPase regulation. Hence we suggest that the high frequencies of certain amino acids at positions x_1_, x_2 _and x_3 _are simply the result of convergent evolution.

Although the amino acid compositions in each position-repeat-type combination show distinct biases, there are also overriding similarities. The analysis below is specific to FliH, but similar biases are seen with YscL. For instance, in the x_1 _position of AxxxG repeats, Arg is found at a much higher frequency (20%) than it is in x_1 _of GxxxG (10%) (Figures 5, 7 and 8). Tyr or Phe account for more than 30% of the residues found in position x_1 _of AxxxG but are never found in positions x_2 _or x_3 _of AxxxG or very rarely for x_2 _or x_3 _of GxxxG. More apparent still is the bias in position x_3 _toward Glu, which accounts for more than a third of the residues found in that position.

In GxxxG repeats, Tyr and Phe account for over 45% of the x_1 _positions, Leu with 15% compared to zero in AxxxG, and then Arg and Lys together making up approximately 15%. Glu, Gln, and Ala together account for about 2/3 of the residues in position x_3_. Of note is that Gln makes up over 15% of the residues in the x_3 _position of GxxxGs, while the similar amino acid Asn, differing from Gln only by virtue of having one fewer methylene group in its side chain, is rarely found in that position.

It is also interesting to examine how the amino acid distribution differs in each of the three repeat types. In general, the amino acid distribution in each repeat position is fairly similar, with a general preference for Ala, Glu, Gln, Arg, Lys, and Tyr. However, there are some obvious differences: AxxxGs and GxxxGs have a very high frequency of Tyr or Phe in position x_1_, whereas these are comparatively rare in GxxxAs. Ala is quite common in position x_3 _of GxxxGs, but is less common in GxxxAs and rare in AxxxGs. Arg is quite common in positions x_1 _and x_2 _in AxxxGs and GxxxAs, but is less common in GxxxGs.

More generally, Figures 7 and 8 suggest that, particularly for GxxxGs, positions x_2 _and x_3 _are basically equivalent in their amino acid preferences, while the amino acid frequencies in position x_1 _are significantly different than that of x_2 _and x_3_. This observation suggests that position x_1 _has a fundamentally different structural role than either positions x_2 _or x_3_; one possibility is that the amino acid in position x_1 _facilitates helix-helix interactions, while the amino acids in x_2 _and x_3 _are involved in maintaining helical stability.

In addition, the frequencies obtained using these FliH and YscL datasets are very similar to those obtained when using sets of sequences where the maximum pairwise identity is 90%, rather than 25%. The frequency distribution for the 25% identity sets depicted in Figures 7 and 8 is also provided for the 90% identity sequence sets in Additional file 4. This observation is consistent with the hypothesis that positions x_1_-x_3 _in the GxxxG repeats have undergone extensive mutation during the course of evolution, but have reached an equilibrium amino acid composition that is consistent with the structural and functional constraints placed on these motifs. That multiple combinations of a few amino acid types are observed, and not a distinct conserved sequence pattern at x_1_-x_3_, suggests that there are multiple permutations of amino acid residues that equally fulfil the structural/functional requirements of these repeats in FliH protein and its role in the flagellar export apparatus.

### Finding correlations between pairs of amino acids in specific positions in the primary repeat segments

We sought to find pairs of amino acids in specific positions that occur together significantly more often than would be predicted by chance. This analysis was performed only for FliH; due to their short primary repeat segments, the same analysis would not be meaningful for YscL proteins. The pair correlation, a value that is greater than one if a particular pair of amino acids in a given pair of positions occurs more often than would be expected by chance, was calculated for each possible pair of amino acids, and in each possible pair of positions, within the primary repeat segments. The statistical significance for each correlation was computed using a χ^2 ^test.

As stated earlier, we hypothesized that certain pairs of amino acids in nearby positions (in the same repeat, or in adjacent repeats) would be significantly correlated, while there would be very few significant correlations, if any, when the positions were farther apart. Table 1 shows the most significant correlations found.

**Table 1:** Significant pair correlations in the FliH glycine repeats

Pattern	n^1^	g^2^	P-value
GxAxGxxxGxAxG	5	4.0	8.0 × 10^-4^

GFxQG	11	2.32	4.0 × 10^-3^

GxxDGFxxG	4	3.73	4.7 × 10^-3^

GHxxGxxxGxAxG	4	3.66	5.5 × 10^-3^

GQxxGYxxG	4	3.41	9.1 × 10^-3^

GxQxGxxQG	5	2.92	1.2 × 10^-2^

GLxxGRxxG	5	2.78	1.7 × 10^-2^

GxxKGxxxGxxxGxxxGxExG	4	2.86	2.8 × 10^-2^

GYxxGFxxG	8	2.01	4.4 × 10^-2^

GYxxGLxxG	8	2.01	4.4 × 10^-2^

GLxQG	7	2.07	4.9 × 10^-2^

Pairs of amino acids that occur together at a significantly higher frequency than would be expected by chance (given their individual frequencies) are shown. ^1^The number of times that this particular pattern occurs. ^2^The number of times more often than would be expected by chance that this pattern occurs. The P-value is the result of a χ^2 ^test; see the experimental procedures section for full details.

As expected, most of the significant patterns found in Table 1 involve residues that are nearby in the primary sequence, although there is an important exception. The most significant correlation is GxAxGxxxGxAxG, which is surprising given that it is a longer-range pattern. It is possible that the Ala residues in the x_2 _positions contribute to helical stability via hydrophobic interactions or by some other mechanism. Some correlations are readily explicable; for instance, the pattern GQxxGYxxG seems plausible, as the NE2 amide hydrogen of the Gln residue at x_1 _should be able to either donate a hydrogen bond to the Tyr residue OH or provide its N-H group to make an amino-aromatic interaction. Furthermore, the NE2 amide hydrogen of a Gln residue in position x_1 _can also donate a hydrogen bond to the backbone carbonyl oxygen of the first Gly residue in the neighbouring twofold related GxxxG helix segment presuming standard GxxxG helix dimerization [26]. However, other patterns are more difficult to explain. For instance, the pattern GYxxGFxxG is found twice as often as would be expected by chance, but the Phe and Tyr side chains are unlikely to interact directly with each other, as both side chains would presumably be in a χ_1 _= 180° conformation favoured by aromatic residues in helices, preventing van der Waals stacking of the aromatic rings. The strong positive correlation may indicate that the combination of these two residues in these positions is conducive to forming helix-helix interactions through close contacts of the aromatic side chain on one helix with the glycine backbone atoms on the adjacent helix, again assuming standard GxxxG helix dimerization.

### Identifying glycine repeats in the helices of other proteins

A set of 7,963 proteins were downloaded from the PDB, and the helices from each protein were examined to determine the presence and length of any glycine repeats. Because GxxxG is the dominant motif in FliH proteins, these helices were examined only for GxxxGs; AxxxGs and GxxxAs were ignored. This analysis is similar to that performed by Kleiger *et al*. [26], who examined another non-redundant PDB set and found that 1.26% of the helices that they examined contained the GxxxG motif. In the present analysis, a total of 85,770 unique helices were examined, and the frequencies of different lengths of glycine repeats are shown in Table 2.

**Table 2:** Glycine repeat frequencies in PDB helices

Repeat	# found	% of all helices
None	84,337	98.3%

GxxxG	1,373	1.6%

GxxxGxxxG	53	0.06%

GxxxGxxxGxxxG	7	0.008%

Longer GxxxG repeats	0	0.0%

A total of 85,770 unique helices from 7,963 PDB proteins were searched for the presence of GxxxG repeats. The number of helices containing a repeat of each length is shown.

The most obvious conclusion that can be drawn from the data in Table 2 is that the long primary repeat segments found in some of the FliH proteins are – at least as far as this dataset is concerned – absolutely unique, which is quite surprising given how nature has a tendency to reuse the same constructs. Information regarding the seven helices that contained a GxxxGxxxGxxxG repeat is provided in Table 3. The amino acids in the variable positions of these repeats are predominantly hydrophobic, and it is obvious that none of these repeat segments are similar to those found in FliH.

**Table 3:** Proteins in the PDB containing the GxxxGxxxGxxxG motif

PDB ID	Helix ID	Repeat
1T5J	1	GSVFGAVIGDALG

1YCE	1	GIGPGVGQGYAAG

2CWC	1	GAFLGLAVGDALG

2CWC	15	GAVYGQLAGAYYG

2D2X	5	GGLTGNVAGVAAG

2FOZ	1	GCLAGALLGDCVG

1NLW	1	GLILGAIVGLILG

Of the 85,770 unique helices examined form PDB entries, just 7 contained the GxxxGxxxGxxxG motif. For each sequence, the corresponding PDB ID is given, along with the identifier of the helix in which the motif is found.

### The structure of glycine repeat-containing helices in other proteins as a model for FliH

Although no crystal structure has been solved for any FliH protein, one can still obtain insight into the structure of the FliH glycine repeats by examining the crystal structures of other proteins that also have glycine repeats. Unfortunately, there are no solved structures of proteins having long glycine repeats. The best alternative would be to use one of the proteins given in Table 3, but unfortunately the amino acid composition of the glycine repeats in these helices is so unlike that of the FliH proteins that none would make a good model for the type of interaction that might be formed between helices in FliH.

Thus, the remaining approach is to find a protein that contains a single GxxxG repeat having FliH-like amino acids in the variable positions. In their analysis of helical interaction motifs in proteins, Kleiger *et al*. [26] provide a table of proteins that contain GxxxG repeats that mediate helix-helix interactions. The glycine repeat in each PDB file given by Kleiger and co-authors was identified, and it was found that some of these contained amino acids in the variable positions that were similar to the amino acids that are commonly found in the glycine repeats in FliH.

**Figure 9:**
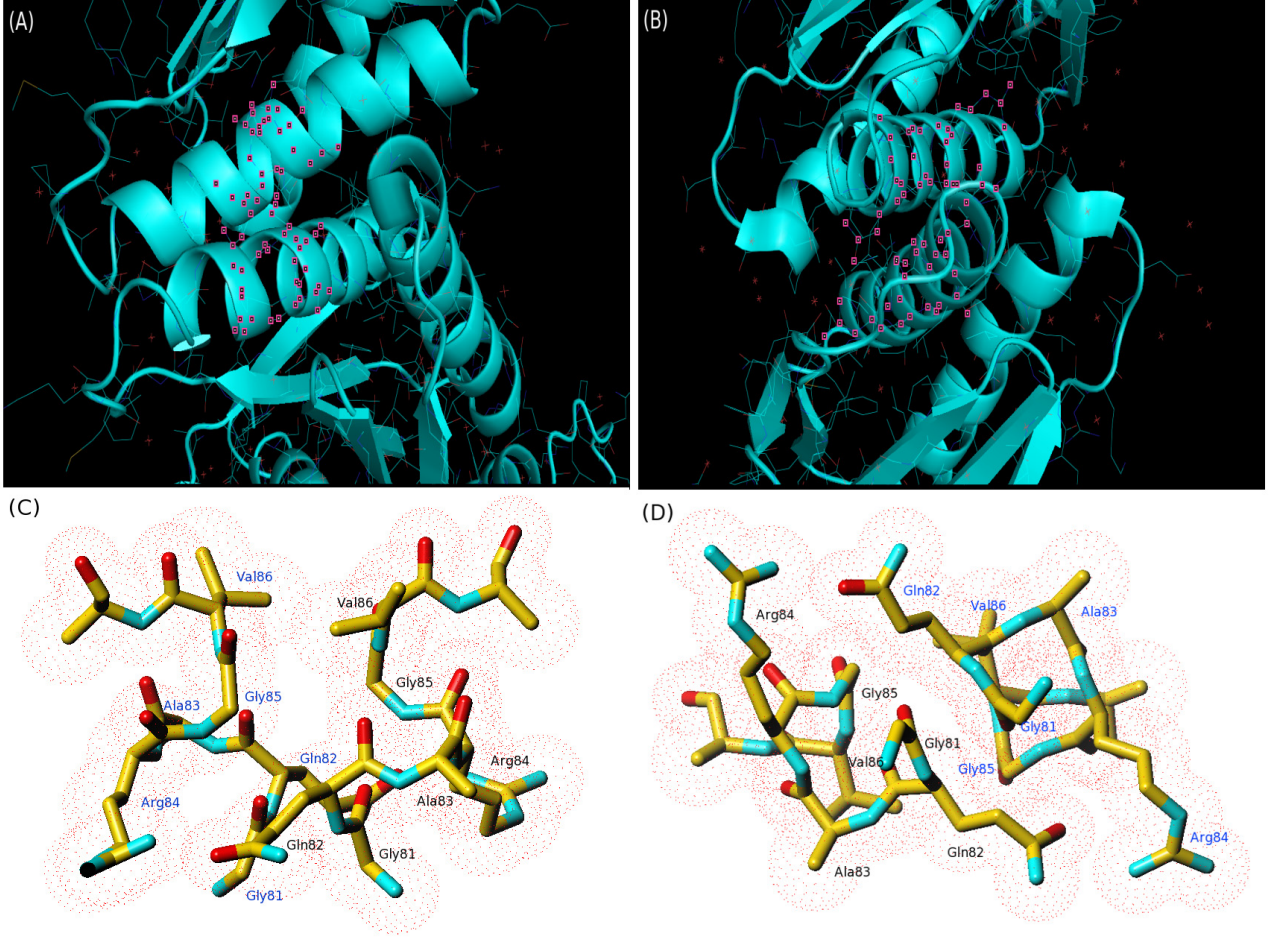
**Glycine repeat-mediated interaction between two helices in *E. coli *site-specific recombinase**. The helix-helix interaction in *E. coli *site-specific recombinase (PDB ID 1HJR) is shown. (A) A side view of the helices that undergo glycine repeat-facilitated dimerization. The pink squares represent the atoms of the residues in the glycine repeat segment. (B) An end-on view of the same interaction. (C) A more detailed representation of the interactions of the individual residues in the glycine repeat, viewed from the side. (D) Detailed representation viewed end-on. (A) and (B) were produced using PyMol [34], while (C) and (D) were produced using TURBO-FRODO [33].

We chose *E. coli *site-specific recombinase (PDB ID 1HJR) as a model for helix-helix dimerization in FliH. This protein contains the glycine repeat GQARG, which – while not the archetypical FliH repeat – contains residues in x_1_, x_2_, and x_3 _that are represented in at least moderate amounts in the same position in FliH repeats. There are proteins given by Kleiger *et al*. that contain repeats with variable amino acids more closely matching those usually found in FliH (1DBT contains the repeat GLEEG, for instance). However, 1HJR was chosen because it features two identical glycine repeat segments (from identical subunits) that dimerize, whereas the helix containing the glycine repeat in 1DBT dimerizes with a helix that does not contain a GxxxG. Given that two FliH proteins dimerize to form a heterotrimeric complex with FliI [17], and that many FliH proteins contain several repeats throughout the protein, it seems likely that, in FliH, dimerization would occur between two helices that both contain glycine repeats, making 1HJR a better model than 1DBT. See Figure 9 for a molecular model of the GxxxG helix-helix dimer in this protein.

Parts (C) and (D) of Figure 9 suggest that interactions between adjacent glycine residues may have an important role in the dimerization process, as the lack of a bulky side chain in this residue allows a C-H... O hydrogen bond to form between the two Gly residues. In addition, the closest contacts between residues with side chains appear to be between the x_1 _position in the first helix and the x_2 _position of the second twofold symmetry-related helix. In the case of 1HJR, the NE of the Arg residue in position x_1 _donates a hydrogen bond to the OE1 oxygen atom of the Gln residue in x_2 _on the opposite helix. Although residues in positions x_2 _and x_3 _can also make interactions with the adjacent twofold symmetry-related helix, they do not appear to be as close together in space.

## Discussion

### Functional significance of the variability in length of glycine repeats in different FliH proteins

Given the large amount of variability in the lengths of the glycine repeat segments in different FliH proteins, it begs the question as to whether helix-helix dimerization or some other property inherent to the GxxxG sequences is functionally important in FliH. If so, it would imply that one of two things is true: either the FliH proteins with few or no glycine repeats are able to form helix-helix dimers anyway, perhaps due to the presence of some other motif, or that these FliH proteins assume some other structure that happens to be functionally equivalent to the helix-helix dimers that are presumably found in the GxxxG repeat-rich FliH proteins. It seems possible that this distinction could be the result of FliH genes ancestrally acquiring a GxxxG segment that has over time undergone convergent evolution, with two or more ancestral proteins evolving semi-independently into a functionally similar end product – some evolving into the glycine repeat-rich FliH proteins, and others evolving into FliH proteins lacking these repeats. The extremely low sequence identity between many FliH proteins would also support this hypothesis. This also raises the question of how such repeats might evolve. Comparison of closely related FliH GxxxG sequence repeats from BLAST searches (results not shown) suggests that additional repeats are likely added one at a time in four residue steps. How this might occur during DNA replication or recombination is not known. The evolution of multiple short sequence motifs, although a challenging problem, is outside the scope of this analysis, but is certain to attract the attention of other researchers in the future.

### Comparison of glycine repeat frequencies with quantitative α-helix propensities

It is interesting to compare the amino acid frequencies given in Figures 7 and 8 with the experimentally-derived propensity of each amino acid to be in an α-helix. The scale derived by Pace and Scholtz [27] assigns a number between 0 and 1 kcal/mol to each amino acid, with higher energies reflecting decreased helix propensity. According to their scale, Ala has the highest helix propensity, while Pro has the lowest. Consistent with this scale, Figures 7 and 8 show that four of the nine position – repeat-type combinations contain Ala at a relatively high frequency (over 10%). In contrast, Leu, the second-most favourable helix-forming residue, is present at high frequencies (~14%) only in position x_1 _of GxxxG repeats. Glu and Gln, which are found at high frequency in the glycine repeats, have only moderate helix propensity according to Pace and Scholtz's scale (lower than Leu, Met, and Lys, all of which are found at much lower frequencies in the primary repeat segments than either Glu or Gln).

It is possible that the amino acid composition required for helix-helix dimerization is distinctly different than that found in a typical α-helix. For instance, we have argued above that the hydrogen bonding capability of side chains (e.g. Glu, Gln, Arg) in positions x_1 _and x_2 _may be very important in side chain-side chain or side chain-backbone interactions in dimeric GxxxG helix-helix interactions. Further work would involve careful structural and biochemical characterization of various idealized GxxxG motifs in peptides and proteins.

It is important to acknowledge that many different scales have been developed for measuring the α-helix propensity of the amino acids, and although they are mostly consistent with one another, each scale is derived from a unique set of experimental parameters. In this case, we have chosen to compare our results with Pace and Scholtz's scale, but other scales are qualitatively very similar, with Ala, Glu, Met, Leu, Phe, Lys and Gln generally acknowledged as being helix forming residues. For instance, one secondary structure propensity scale that is commonly found in biochemistry textbooks lists Glu as the most favorable helix residue, which is more consistent with the composition of the glycine repeats in FliH. However, this same scale also lists Tyr as being somewhat unfavourable in helices, whereas in FliH Tyr is strongly favoured in position x_1 _of AxxxG and GxxxG motifs. This underscores the often stated caveat that context is everything in protein structure. The presence of glycine in such helical segments reinforces this point, as glycine residues are not normally acknowledged as being helix formers except within certain local sequence contexts.

### Looking beyond the PDB to find proteins with glycine repeats

We report that there are no sequences found in the PDB set that we downloaded containing helices with glycine repeats anywhere near the length of those found in some FliH proteins. As a relatively small fraction of all known protein sequences have had their structures solved, one would have a better chance of finding long glycine repeats by searching a larger database of protein sequences (not structures), such as the Swiss-Prot database. Some preliminary analysis was performed as a starting point for addressing this problem. The entire Swiss-Prot database, which consisted of 261,515 sequences at the time that it was downloaded, was searched for FliH-like glycine repeat segments. Of course, since these sequences do not contain secondary structure information, there was no way to limit the search to α-helices. Eighteen sequences were found that contained repeat segments of length 11 or longer; however, all of these segments consisted of low-complexity repeats (for instance, the protein with Swiss-Prot accession number P19260 contains the repeat GSAGGSAGGSAGGSAGGSAGGSAGGSAGGSAGGSAGGSAGGSAGGSAGG), and thus were in no way analogous to repeats in FliH. The longest glycine repeat segment that was not a low-complexity repeat was of length 10, which was found in a presumably uncharacterized protein from *Rickettsia japonica *simply called "17 kDa surface antigen" (Swiss-Prot accession number Q52764). Further analysis would have to be done with this Swiss-Prot-derived sequence information in order to identify repeat segments that are similar to those found in FliH.

## Conclusion

While many different short protein sequence motifs have been characterized, the glycine repeats in FliH and YscL are an unusual example. Firstly there is an obvious structural hypothesis to put the general features of the sequence motif in context and amino acid secondary structure preferences for residues found in the repeats strongly suggest an α-helical structure. However, not all observed pairwise residue correlations in adjacent repeats are entirely well-explained within the context of the presented structural model. In addition we have no plausible explanation for why only FliH proteins, and no other sequences, contain these unique GxxxG repeats. There is also no obvious reason or explanation for the highly variable number of repeats in different FliH sequences. However, sequence deletions in *Salmonella *FliH that affect *in vitro *ATPase hydrolysis assays for a FliI:FliH complex (either by enhancing or reducing FliI's ATPase activity) overlap with one or more of the *Salmonella *FliH GxxxG repeats (see introduction) [17]. This suggests that secondary interactions between FliI and FliH, in addition to the well-known interaction between the C-domain of FliH and the N-terminal 15 residues of FliI, may depend critically on the presence of the GxxxG motif [15,18]. Studies on the ATPase activities and/or export capability of FliI:FliH pairs from other motile bacteria with engineered deletions in the FliH GxxxG repeats would likely shed light on the importance of the GxxxG repeats in flagellar protein export. While the extremely long length of the repeats in some FliH proteins implies that the repeats may cooperate to perform an important functional or structural role, the fact that other FliH sequences have short repeats segments, or even no repeat segment at all, would suggest otherwise. Alternately, another unidentified protein involved in the flagellum export pathway may be able to compensate for deletion of the GxxxG motifs in FliH. Given the likely structural constraints on FliH participating in the flagellar export pathway via interactions with FliI, FliN and other proteins at the base of the flagellar export pore, it will be interesting to see if more than one protein participates in interactions with the FliH GxxxG motifs. It is also interesting that extremely long glycine repeats evolved in FliH, but not in its Type III secretion homologue YscL, and this may actually tell us something, albeit cryptically, about differences in the two export systems. The extremely biased amino acid composition of the glycine repeats suggests that these regions may adopt nonstandard helix-helix tertiary or quaternary interactions that will be of interest for structural biologists to elucidate. Lastly, and perhaps most interestingly, the extreme rarity of this motif in other proteins is very surprising given that nature tends to find similar structural solutions to a biological problem multiple times. Crystal structures and careful biochemical/biological analysis of these proteins should ultimately be able to address these fascinating issues.

## Methods

### Acquiring the set of FliH proteins

We endeavored to acquire FliH proteins from as many different bacterial species as possible. To accomplish this, GenBank was searched for protein sequences whose annotation contained the word "FliH", and these protein sequences were downloaded. In addition, the FliH sequence from *Salmonella *and the FliH sequence was *H. pylori *were used as input to PSI-BLAST, and the sequences attaining e-values of less than 10^-3 ^after two iterations were downloaded. All of these sequences were aggregated into a single set that will be denoted "set A".

### Filtering of FliH sequences

Redundancy in set A was reduced by using the EMBOSS [28] program *needle *to perform pairwise global alignments [29] between all possible pairs of sequences. That is, each sequence in set A was globally aligned with every other sequence, and the % identity between each pair of sequences was recorded. The gap opening penalty used in *needle *was 8, while the gap extension penalty was set to 0.5; all other settings were left at their default values. Using the % identity data for each pair in set A, a new set of proteins ("set B") was derived such that no protein in the latter set was more than 25% identical to any other protein in that same set. The purpose of this was to eliminate as much as possible the phylogenetic signal, which could potentially confound the statistical results. This set was used to derive the data shown in Figures 4, 5, 7 and 8. For comparison purposes, a larger set of proteins was created; in this set, no protein was more than 90% identical to any other protein. Analysis of this set is shown in Additional files 3 and 4.

Note that the obvious method for deriving set B is simply to randomly delete one of the proteins whenever two proteins in set A are found to be more than 25% identical. However, this method may result in more proteins being deleted than necessary; consider three proteins X, Y, and Z, and that proteins X and Y are both more than 25% identical to protein Z, but are not more than 25% identical to each other (casual testing suggested that this does happen occasionally). Suppose that X is first compared to Z and found to be more than 25% identical, and X is arbitrarily chosen for deletion. Then Y is compared to Z, and one of these proteins is deleted. Now only one protein is left, despite the fact that only Z needed to be deleted in order to satisfy the requirements of set B. To solve this problem and maximize the number of sequences left after filtering, the following algorithm was used: for each protein *p *in set A, a set ψ_*p *_is maintained that contains all the other proteins that are more than 25% identical to *p*. The sequence *M *with the highest value of |ψ_*M*_| is found, and *M *is then removed from set A; in addition, *M *is also deleted from every other protein's ψ_*p*_. This process is repeated until ψ_*p *_= ∅ for all *p*.

To remove proteins that were unlikely to actually be FliH, the mean length μ of the sequences in set B was computed, as well as the standard deviation σ of these lengths. Protein sequences having a length outside the range μ ± 1.5σ were deleted. Finally, a multiple alignment of the sequences was created using T-coffee [30], and sequences were deleted that, based on the alignment, looked as if they were unlikely to actually be FliH.

### Acquiring and filtering the YscL sequences

The procedure used to acquire YscL sequences was similar to that used to acquire the FliH sequences. The only difference was that, due to their inconsistent naming conventions, a GenBank search was not performed; rather, the set consisted only of significant matches from a PSIBLAST search using the YscL sequence from *Yersinia enterocolitica*. The sequences were then filtered in the same manner as the FliH sequences.

### Characterization of amino acid frequencies in the primary repeat segments

A Perl script was written to determine, for each repeat type, the frequency by which each amino acid is found in positions x_1_, x_2 _and x_3_. Only repeats in the primary repeat segments were analyzed; repeats in secondary repeat segments were ignored. To ascertain whether the amino acid distribution in each position–repeat-type combination was significantly different than the overall amino acid composition of FliH proteins, the mean frequency of each amino acid in the FliH proteins was computed, and this was compared (separately) to each of the amino acid distributions described above by using a χ^2 ^test. Let *E*_*ikR *_denote the number of times that amino acid *i *would be expected to be found in position x_*k *_of repeat type *R *given the overall frequency of *i *in the entirety of the FliH proteins. That is, *E*_*ikR *_is equal to the fraction of residues in the FliH proteins that are amino acid *i*, multiplied by the total number of repeats of type *R*. If *O*_*ikR *_denotes the observed count, then under the null hypothesis (*E*_*ikR *_= *O*_*ikR *_for each amino acid *i*),

is distributed as χ^2 ^with 19 degrees of freedom. The P-value corresponding to each χ_kR_^2 ^was determined using the Statistics::Distributions Perl module.

### Determining correlations between pairs of amino acids in the primary repeat segments

To determine whether certain pairs of amino acids occur together in certain positions at frequencies significantly greater than would be expected by chance, correlations for all possible pairs of amino acids were calculated for each possible pair of positions within a given primary repeat segment. Correlations were determined only in GxxxG repeats (AxxxGs and GxxxAs were ignored). Statistical analysis was performed as described previously [31,32]. Consider a typical segment in a FliH protein with *m *GxxxG repeats. Define *n*_*ijkld *_to be the number of times that amino acid *i *is found at position x_*k *_in some arbitrary repeat *r *(1 ≤ *r *≤ *m*), *and *amino acid *j *is found at position x_*l *_in the (*r *+ *d*)^th ^repeat (1 ≤ r + d ≤ m). Thus, the possible values for *i *and *j *are the 20 amino acids, and *k *and *l *can each be either 1, 2, or 3. Values for *d *range from 0 to 9; the upper value was chosen because the longest repeat found in any FliH protein in set B was of length 10. If *d *= 0, then this means that the two amino acids in the pair are in the same repeat; if *d *= 1, it means that they are in adjacent repeats, and so on. When *d *= 0, *k *<*l*. To compute *n*_*ijkld*_, the following procedure was used:

For each FliH sequence *p*

   For each GxxxG repeat *r *in *p *with *r *+ *d *≤ *m*

      If position x_*k *_in repeat *r *contains residue *i and*

      position x_*l *_in repeat (*r *+ *d*) contains residue *j*

         Add 1 to *n*_*ijkld*_

The expected value of *n*_*ijkld*_, assuming that no correlation exists, is

where  is the number of times amino acid *i *is found at position x_*k *_(with any amino acid at position x_*l*_),  is the analogous value for the other amino acid, and  is the total number of pairs. Note that superfluous subscripts are dropped in the preceding notation.

Finally, let

denote the pair correlation, which will be greater than one if the amino acids at the indicated positions are found at a greater frequency than would be expected given their individual frequencies in those positions, and vice versa.

The significance of each correlation was computed using a χ^2 ^test:

If the null hypothesis is true (*n*_*ijkld *_= *E*_*ijkld*_), then χ^2^_*ijkld *_will have a χ^2 ^distribution with one degree of freedom.

The following is an example to illustrate the above procedure. Assume that we want to find the pair correlation between Asp in position x_3 _and Glu in position x_1 _in pairs of repeats that have one repeat between them. This corresponds to the pattern GxxDGxxxGExxG, and therefore *i *= D, *j *= E, *k *= 3, *l *= 1, and *d *= 2. Also assume that the number of possible instances in which these amino acids could occur together in the stated pattern, in all the FliH proteins, is 263 (n_*d *_= 263). Of these instances, Asp is found in position x_3 _of the left-hand repeat 22 times, while a Glu occurs in position x_1 _of the right-hand repeat 9 times (*n*_*ikd *_= 22 and *n*_*jld *_= 9). Thus, the number of times you would expect Asp and Glu to appear together in these positions, assuming no correlation, is *E*_*ijkld *_= (22 × 9)/263 = 0.753. The actual number of times that they occur together is *n*_*ijkld *_= 5; the pair correlation is thus *g*_*ijkld *_= 5/0.753 = 6.64, meaning that this pairing of amino acids in the stated positions is found 6.64 times as often as would be expected at random. The χ^2 ^value is (5 - 0.753)^2^/0.753 = 23.95, which corresponds to a P-value of 9.8 × 10^-7^, meaning that this correlation is certainly statistically significant.

### Identifying glycine repeats in proteins in the Protein Data Bank

7,963 proteins were downloaded from the PDB by first searching for molecules that contain protein, then removing structures solved by a method other than X-ray crystallography, and finally using the "remove similar sequences at 40% identity" option.

Each PDB file was searched using a Perl script for helices that contain glycine repeats. If multiple helices had the exact same sequence, then all but one of these were discarded. This occurred both in the same protein (when there are multiple identical subunits), and between proteins (despite the sequences being less than 40% identical according to the PDB's criteria, some PDB files still contained helices with sequences that were the same as helices found in another PDB file).

### Protein visualization

TURBO-FRODO [33] and PyMol [34] were both used as protein visualization tools.

### Secondary structure prediction

The tools in references [35-39] were used for secondary structure predictions of the GxxxG repeats and those shown in Figures 1, 2 and 3.

## Authors' contributions

BT devised and implemented the database extraction procedures and the statistical tests. SM identified the FliH repeats and preliminary statistical preferences for positions x_1 _to x_3_. Both authors contributed to the writing of the manuscript and in preparation of figures. Both authors read and approved the final manuscript.

## Supplementary Material

Additional file 1 Fasta-format FliH sequences filtered using a 25% sequence id cutoff filter, used for the analysis. Click here for file

Additional file 2 Aligned set of FliH sequences at 25% sequence id cutoff output from T-Coffee Click here for file

Additional file 3 Histogram of the number of sequences containing a given number of repeats for FliH at a 90% sequence id cutoff. Click here for file

Additional file 4 Amino acid frequency histograms for positions x_1_, x_2 _and x_3 _for each of the repeat types in FliH and YscL sequences at 90% id cutoff criteria. Click here for file
