# Supplementary figures and images for: Statistical characterization of the GxxxG glycine repeats in the flagellar biosynthesis protein FliH and its Type III secretion homologue YscL
Source: BMC Microbiol. 2009 Apr 16;9:72. doi: 10.1186/1471-2180-9-72 (PMC2674601; doi:10.1186/1471-2180-9-72)

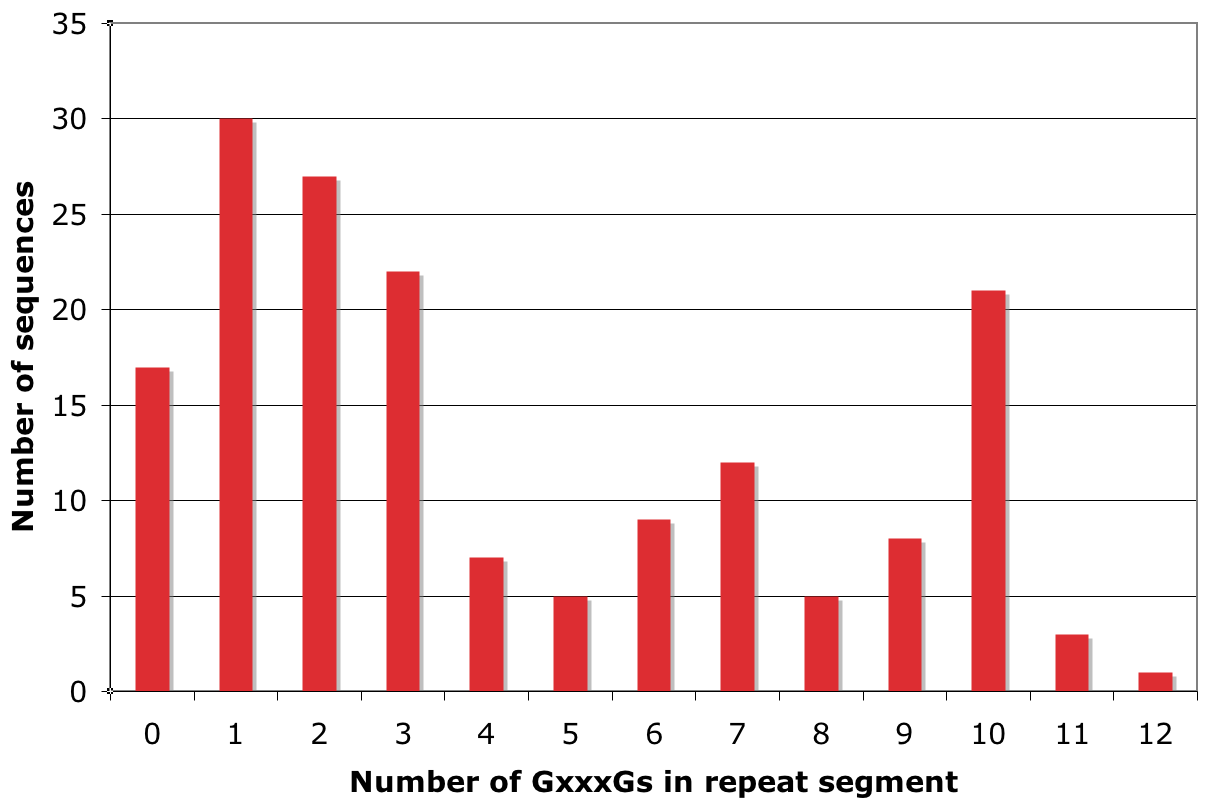

Supplement: Additional file 3 — Histogram of the number of sequences containing a given number of repeats for FliH at a 90% sequence id cutoff. [file 1471-2180-9-72-S3.png]

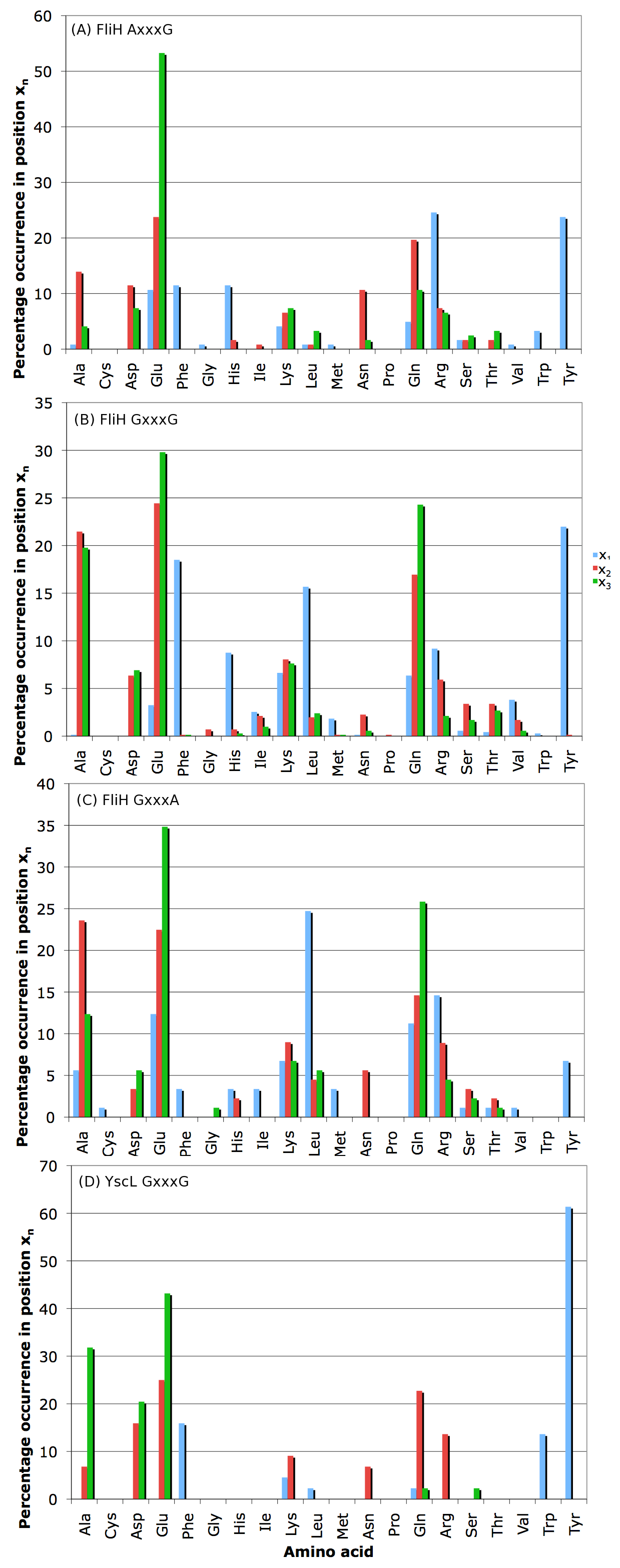

Supplement: Additional file 4 — Amino acid frequency histograms for positions x1, x2 and x3 for each of the repeat types in FliH and YscL sequences at 90% id cutoff criteria. [file 1471-2180-9-72-S4.png]
